# The role of PD-L1 in the immune dysfunction that mediates hypoxia-induced multiple organ injury

**DOI:** 10.1186/s12964-021-00742-x

**Published:** 2021-07-13

**Authors:** Yang Sun, Jin Tan, Yuyang Miao, Qiang Zhang

**Affiliations:** 1 Department of Geriatrics, Tianjin Medical University General Hospital, Tianjin Geriatrics Institute, Anshan Road NO.154, Tianjin, 300052 China; 2 Tianjin Medical University, Tianjin, China

**Keywords:** Hypoxia, Multiple organ injuries, Immune dysfunction, PD-1, PD-L1

## Abstract

Hypoxia is a pathological condition common to many diseases, although multiple organ injuries induced by hypoxia are often overlooked. There is increasing evidence to suggest that the hypoxic environment may activate innate immune cells and suppress adaptive immunity, further stimulating inflammation and inhibiting immunosurveillance. We found that dysfunctional immune regulation may aggravate hypoxia-induced tissue damage and contribute to secondary injury. Among the diverse mechanisms of hypoxia-induced immune dysfunction identified to date, the role of programmed death-ligand 1 (PD-L1) has recently attracted much attention. Besides leading to tumour immune evasion, PD-L1 has also been found to participate in the progression of the immune dysfunction which mediates hypoxia-induced multiple organ injury. In this review, we aimed to summarise the role of immune dysfunction in hypoxia-induced multiple organ injury, the effects of hypoxia on the cellular expression of PD-L1, and the effects of upregulated PD-L1 expression on immune regulation. Furthermore, we summarise the latest information pertaining to the involvement, diagnostic value, and therapeutic potential of immunosuppression induced by PD-L1 in various types of hypoxia-related diseases, including cancers, ischemic stroke, acute kidney injury, and obstructive sleep apnoea.

**Video Abstract**

**Supplementary Information:**

The online version contains supplementary material available at 10.1186/s12964-021-00742-x.

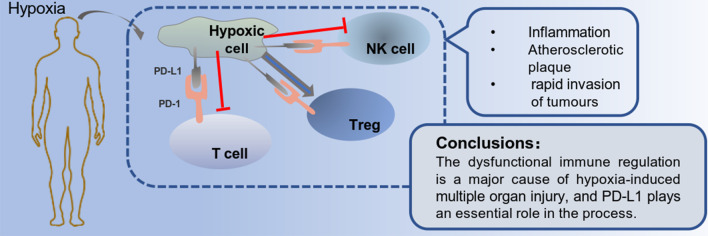

## Background

As the primary pathological mechanism involved in cancer, ischemic stroke, acute kidney injury (AKI), obstructive sleep apnoea hypopnoea syndrome (OSAHS), and many other diseases, hypoxia can cause disease progression by affecting the cell cycle, metabolism, autophagy, apoptosis, and other cellular mechanisms [1–3]. However, many complications associated with hypoxia occur through unknown mechanisms, such as OSAHS-related heart damage and multiple organ failure caused by tumours. With the increasing number of studies investigating the harmful mechanisms of hypoxia and the adaptations which occur in response to these diseases, it has been shown that hypoxia regulates immune responses, and this dysfunction plays an essential role in the secondary destruction of organs and systemic complications [4, 5]. Despite the growing knowledge about the effect of hypoxia on the immune function and the role of the immune response in several clinical diseases, there is no clear consensus on the precise effects of immune system dysfunction in hypoxia-induced multiple organ injury.

Programmed cell death protein 1 (PD-1) and its endogenous ligand, programmed death-ligand 1 (PD-L1), are essential immune checkpoint molecules that modulate apoptosis [6]. In combination with PD-L1, PD-1 regulates the immune system through promoting the differentiation of regulatory T cells (Tregs) and initiating T cells apoptosis [7, 8]. In isolation, this mechanism would result in a reduction in autoimmune diseases; however, the capability of immune cells to kill tumour cells becomes simultaneously suppressed. Over time, researchers have found that the pathway of PD-1/PD-L1 also plays an essential role in the resolution of stroke-related neuroinflammation, the decrease in immune function that occurs in patients with OSAHS, and the inflammation that occurs in AKI.

In recent years, studies have shown increased levels of PD-L1 expression in various experimental models of hypoxia and in patients exposed to hypoxic conditions. Furthermore, the PD-1/PD-L1 pathway is extensively involved in certain pathophysiological processes of hypoxia-related diseases by promoting the differentiation of Tregs and suppressing the function of T cells [7–9]. However, the specific role and mechanism of action of PD-L1 in different diseases are not fully understood, and the potential relationship between hypoxia and PD-L1-mediated signalling remains elusive. In this review, we aimed to summarise the known interactions between hypoxia and the immune system, the possible mechanisms responsible for the upregulation of PD-L1 induced by hypoxia and its effect on immune function, and the latest information pertaining to the involvement and therapeutic potential of the PD-1/PD-L1 signalling in different hypoxia-related diseases. This article mainly focuses on determining whether PD-L1-dependent immunosuppression is related to the development of hypoxia-induced multiple organ injury and the various roles it plays in different conditions.

## The interaction between hypoxia and immune function

**Fig. 1:**
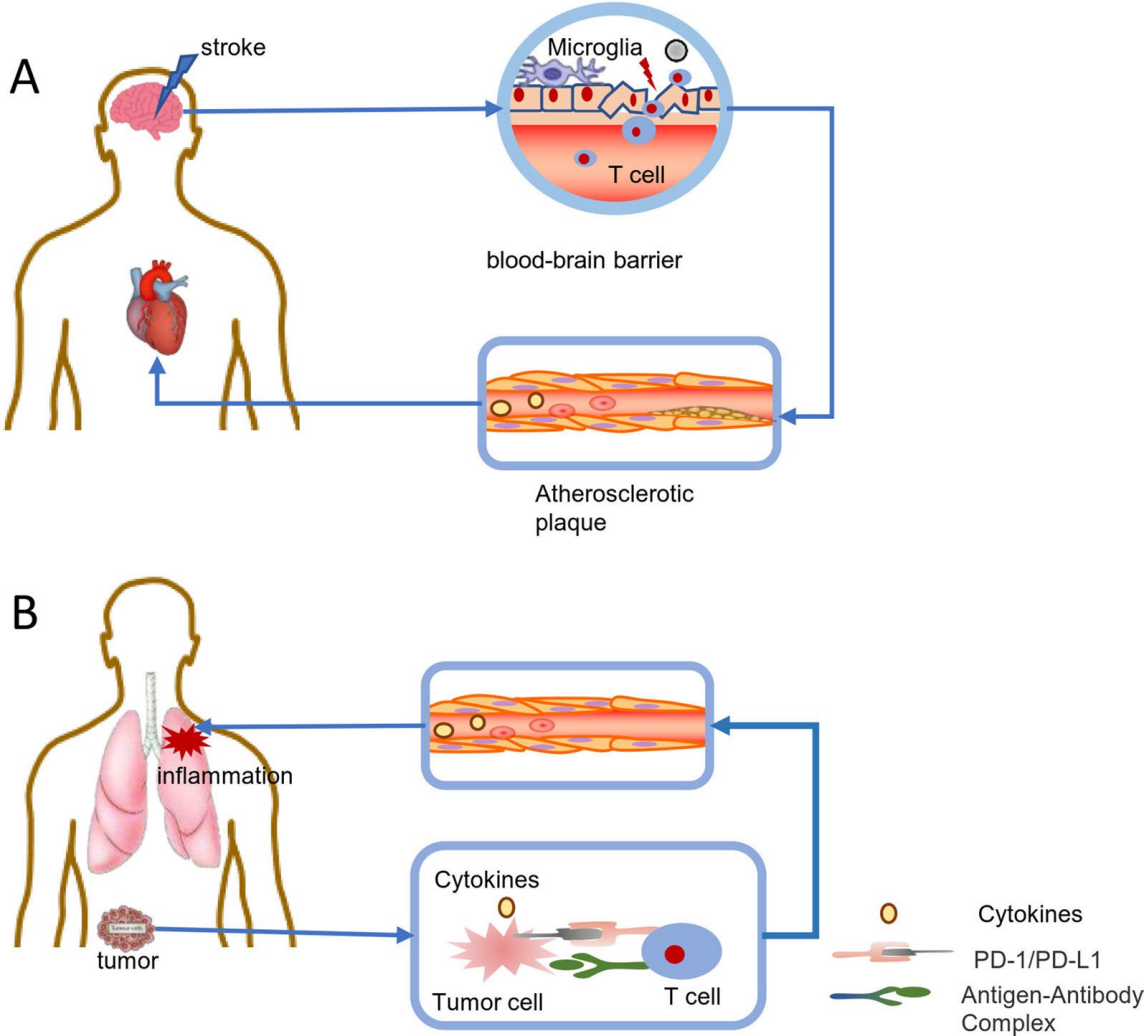
The role of immune system dysfunction in hypoxic diseases. **A** Immune system dysfunction mediates ICH-induced cardiac dysfunction. After stroke, the release of cytokines by activated microglia damages the blood–brain barrier, permitting cytokine entry into the periphery to activate the peripheral immune system, causing further damage to the heart and other organs. **B** The role of immune system dysfunction in cancer progression. Tumour cell antigens can be distinguished by T cells, while increased expression of PD-L1 and PD-1 leads to further inhibition of T cell function. Abbreviations: *ICH* intracerebral haemorrhage, *PD-L1* programmed death-ligand 1, *PD-1* programmed cell death protein 1

In clinical practice, hypoxia, especially severe hypoxia, accompanied by multiple organ failure is often associated with serious complications and a poor prognosis. In many diseases, multiple organ dysfunction is often associated with disordered immune and inflammatory responses. Unlike the direct tissue damage caused by hypoxia, hypoxic injury may induce functional changes of the immune system leading to secondary injuries. Meanwhile, inflammatory changes may also aggravate hypoxia-induced tissue damage. For example, in coronavirus disease 2019 (COVID-19), cardiac injuries and AKIs often do not occur as a direct result of viral infection, but instead result from damage caused by inflammatory responses, which reduce oxygen intake and injure endothelial cells, leading to intravascular coagulation and thrombosis [10]. In tumours, the hypoxic environment inhibits the immune surveillance function of T cells, promotes the rapid growth of tumours, and increases the incidence of infection [11–13]. The release of cytokines from activated microglia following a stroke causes neuroinflammation, aggravating the cerebral infarction area and damaging the blood–brain barrier. The damaged blood–brain barrier results in those cytokines entry into the periphery where they activate the peripheral immune system and cause damage to the heart and other organs [14, 15]. Systemic inflammatory and immune responses caused by sleep apnoea may be relevant risk factors for the development of atherosclerosis (Fig. 1) [16–19]. From this perspective, the differential effects of hypoxic diseases on immune function can be somewhat tissue-specific, either stimulating inflammation or inhibiting immunosurveillance. The disastrous outcomes of these disorders of the immune system include systemic organ damage and multiple organ failure.

**Fig. 2:**
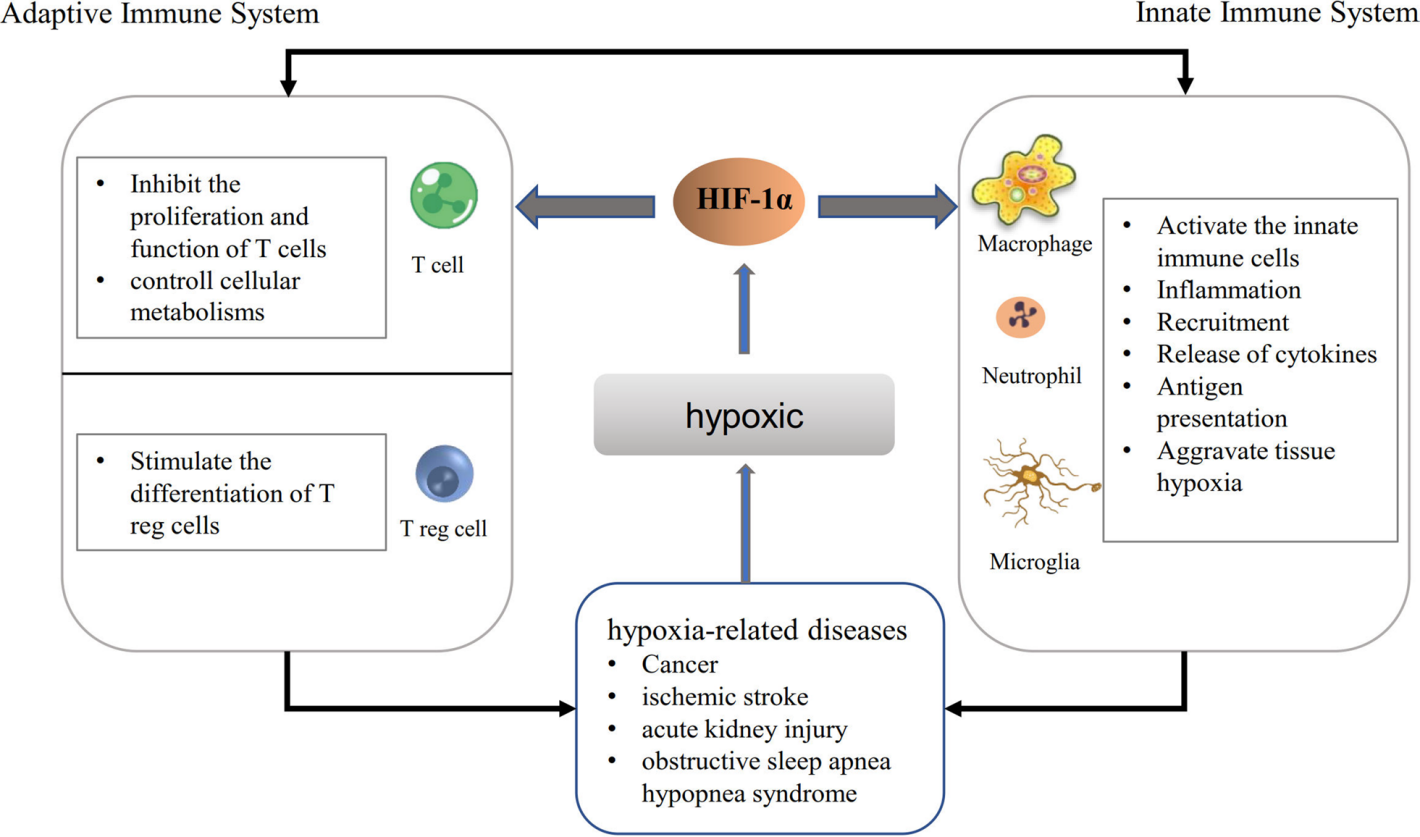
Hypoxia-induced changes in the immune system. Activation of the HIF pathway activates innate immune cells and amplifies the inflammatory response in the hypoxic environment. Meanwhile, the activated HIF pathway suppresses the response of the adaptive immune system by inhibiting the proliferation and function of T cells and stimulating the differentiation of Treg cells. Abbreviations: *HIF* hypoxia-inducible factor, *Treg* regulatory T

How does the hypoxic environment affect the immune system? Generally, hypoxia activates innate immune cells, such as macrophages, neutrophils, dendritic cells, and natural killer (NK) cells, which can rapidly eradicate pathogens. In contrast, adaptive immunity is suppressed under hypoxic conditions, as manifested by the stimulated differentiation of Tregs and the negative regulation of CD4^+^ helper T (Th) cells and CD8^+^ cytotoxic T cells [5]. Mechanistically, the most crucial factor involved in these changes is the activation of hypoxia-inducible factor (HIF) [20, 21]. The activated HIF pathway regulates the immune cells function by modulating cellular metabolic pathways, such as those related to glycolysis and amino acid metabolism [22, 23]. HIF can also bind to the transcription factor forkhead box P3 (FOXP3) and activate the thymus-specific isoform of RAR-related orphan receptor gamma (RORγt), thereby regulating the equilibrium between Th17 cells and Tregs [24]. On the other hand, hypoxia can induce the release of the contents of necrotic cells, cytokines, and immune metabolites from inflammatory sites in tissues. Once released, these active substances and necrotic cell contents not only activate the HIF pathway of immune cells and exert functional autoinhibitory effects, but they also stimulate inflammatory reactions of immune cells directly by acting as antigens and foreign bodies. In addition, when cells are injured by hypoxia, they release more extracellular vesicles, which target immune cells such as effector T cells, NK cells, and monocytes, while inducing the Tregs differentiation and inhibit the immunologic response [25–27]. These examples highlight the fact that the hypoxia-induced regulation of immune function is extremely complicated, and many other immune-related signalling pathways and intracellular molecules will be affected by the hypoxic environment, such as high-mobility group box protein 1 (HMGB1) and Toll-like receptor 4 (TLR4), among others [28, 29]. Most of the previous studies investigating the function of hypoxia-suppressing immune cells have focused on the metabolic and pro-apoptotic pathways (Fig. 2). In this review, we focus on the immune checkpoint: PD-L1, a relatively novel target involved in hypoxia-related immune dysfunction in various hypoxic diseases.

## Effect of hypoxia on the expression of PD-L1

### The expression of PD-L1 is upregulated in various models of hypoxia

PD-L1 has recently attracted much attention for its effect in inhibiting the proliferation and function of T cells, leading to the immune escape of tumours. However, we found that, except for tumours, PD-L1 is also overexpressed in other hypoxia-related diseases such as stroke, AKI, myocardial infarction, and obstructive sleep apnoea, and its immunosuppressive features are linked to disease progression.

PD-L1 is expressed in many non-haematopoietic cells, such as cancer cells, microglia, astrocytes, neurons, and epithelial cells. Several other non-lymphoid cells, such as those of the muscle, heart, placenta, and renal tubular cells, also express PD-L1. Pathological hypoxia can induce the overexpression of PD-L1. For example, PD-L1 is upregulated in tumour cells and tumour-infiltrating myeloid cells [30]. PD-L1 has been shown to be overexpressed in the spleen and central nervous system (CNS) post-stroke in murine models [31]; similar changes in PD-L1 expression have been reported in monocytes from OSA patients [32, 33]. These findings suggest that both persistent and intermittent hypoxia can directly trigger the overexpression of PD-L1 in various cell types. Hypoxia also stimulates the release of exosomes expressing PD-L1, which can reduce cytokine levels and induce T cell apoptosis.

### The mechanism of hypoxia-induced PD-L1 upregulation

#### Hypoxia regulates PD-L1 mRNA expression by activating the transcription factors HIF-1α and NF-κB

Transcriptional factors, such as HIF-1α, and nuclear factor-κB (NF-κB), can regulate PD-L1 expression through targeting its promoter region [30]. The upregulated PD-L1 induced by hypoxia is generally related to changes in HIF-1α [34], and many related anti-tumour drugs inhibit PD-L1 expression by downregulating HIF signalling [35]. Noman et al. previously confirmed that HIF-1α could regulate both the mRNA and protein expression of PD-L1 by directly acting on hypoxia response element 4 (HRE-4) in the proximal promoter of PD-L1 in tumour models [30]; it was shown that inhibition of HIF-1α activity under hypoxic conditions significantly decreased PD-L1 mRNA and protein levels. Likewise, HIF-1α activity also regulates PD-L1 expression in monocytes in patients with sepsis and obstructive sleep apnoea [33, 36]. These indicate that a high level of HIF-1α induced by hypoxia can result in the overexpression of PD-L1 and lead to the inhibition of immune function.

Moreover, NF-κB, which is strongly activated by the presence of cytokines or bacterial products at the site of inflammation, upregulates the PD-L1 indirectly by promoting the transcription of HIF-1α mRNA [4]. Furthermore, NF-κB can directly regulate the expression of PD-L1 [37]. The overexpression of PD-L1 induced by interferon-γ (IFN-γ) is dependent on NF-κB activity. The application of autophagy inhibitors results in the activation of NF-κB in tumour cells, leading to PD-L1 upregulation. NF-κB also contributes to the maintenance of a stable level of PD-L1 and helps inhibit its ubiquitination and degradation, which is the foundation for stabilising the PD-L1 expression induced by tumour necrosis factor-α (TNF-α) in cancer cells.

#### Cytokines released from damaged tissue or inflammatory sites induce the expression of PD-L1

Interestingly, when the hypoxic environment induces cellular damage, chronic inflammation, or the activation of immune cells, various cytokines are released, such as TNF-α and IFN-γ; in turn, these cytokines inhibit immunological functions by upregulating the PD-L1 expressed on target cells. In murine models, the depletion of IFN-γ can lead to decreased PD-L1 in tumour cells [38]. Similarly, in antigen-presenting cells (APCs), treatment with unstimulated monocytes failed to induce a response, whereas a rapid upregulation of PD-L1 was found after IFN-γ stimulation [39]. TNF-α, analogously, can stimulate the upregulation of PD-L1 by the modulation of NF-κB signalling in tumour cells which are undergoing epithelial–mesenchymal transition [40]. In addition, cytokines produced by infiltrating immune cells, such as interleukin 10 (IL-10), interleukin 4 (IL-4), and bacterial lipopolysaccharide (LPS), may also influence the expression of PD-L1 [41].

#### Extracellular vesicles released in the hypoxic microenvironment can play an immunosuppressive role by modulating PD-L1 expression

Hypoxia is believed to alter both the quantity and contents of tumour-derived exosomes (TEXs), and TEXs can induce PD-L1 expression in the lipid bilayer of nanovesicles [42]. For instance, metastatic melanomas release exosomes that transport PD-L1 on their surface [43]. In the hypoxic environment, TEXs enhance the inhibitory effects of myeloid-derived suppressor cells (MDSCs) on gamma delta T (γδT) cells through the regulation of microRNA-21/PTEN/PD-L1 [44]. Besides, there are also some changes in exosomes and microsomes in OSA and stroke mice models, but the relationship between them and PD-L1 is still under investigation.

## PD-L1 suppresses immune function in hypoxic diseases via binding to PD-1

### General mechanism and effects of the PD-1/PD-L1 pathway

**Fig. 3:**
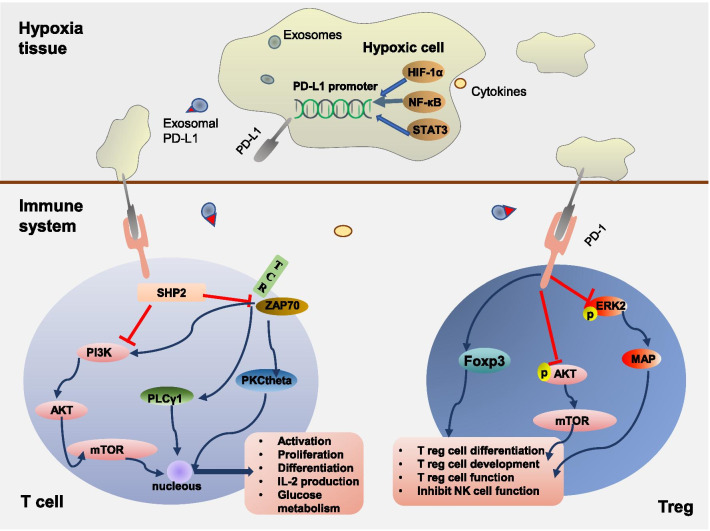
Overexpression of PD-L1 in hypoxic cells and the mechanism of the PD-1/PD-L1 signalling pathway. Hypoxia can contribute both directly and indirectly to the upregulation of transcription factors, including HIF-1α, NF-κB, and STAT3, which act on the promoter of PD-L1 to regulate its expression. At the same time, the cytokines released by hypoxic cells or immune cells can stimulate the expression of PD-L1. In hypoxic cells, extracellular vesicles transporting PD-L1 on their surface are released. Mechanistically, the PD-1–PD-L1 complex modulates immune dysfunction by binding to TCRs and momentarily associating with the phosphatase SHP2. This results in the dephosphorylation of proximal TCR signalling molecules, such as ZAP70, and the decreased phosphorylation of TCR downstream signalling molecules like PLCγ1 and PKCtheta, similar to the role that inhibition of the PI3K/Akt pathway plays in these processes. Therefore, PD-1–PD-L1 binding affects the activation, proliferation, differentiation, metabolism, and IL-2 production of T cells. Furthermore, the PD-1/PD-L1 pathway promotes differentiation and development, sustaining the function of regulatory T cells by enhancing Foxp3 expression, inhibiting the Akt/mTOR signalling pathway, and attenuating the phosphorylation of ERK2. And the activation of Tregs participate in the PD-1–PD-L1 axis-mediated NK cells dysfunction. Abbreviations: *PD-L1* programmed death-ligand 1, *PD-1* programmed cell death protein 1, *HIF-1α* hypoxia-inducible factor 1 alpha, *NF-κB* nuclear factor kappa B, *STAT3* signal transducer and activation of transcription-3, *TCR* T cell receptor, *SHP2* Src homology 2 domain-containing tyrosine phosphatase 2, *ZAP70* zeta chain of T cell receptor-associated protein kinase 70, *PLCγ1* phospholipase C gamma 1, *PKCtheta* protein kinase C theta, *PI3K* phosphoinositide 3-kinase, *AKT* protein kinase B, *IL-2* interleukin 2, *mTOR* mechanistic target of rapamycin, *ERK2* extracellular signal-regulated kinase 2, *Treg* regulatory T

As mentioned earlier, when cells are exposed to hypoxic conditions, more PD-L1 is expressed on the cell surface, and exosomes containing PD-L1 are released. To exert its effects, PD-L1 combines with its target receptor PD-1. The PD-1/PD-L1 pathway inhibits the activity of T cells and promotes the development of Tregs, induces apoptosis in antigen-specific T cells, and inhibits apoptosis in Tregs [45]. Moreover, binding to PD-L1, the activated phenotype marked by expressing PD-1 on NK cells is suppressed [46]. Therefore, the PD-1/PD-L1 pathway plays an important role in inhibiting the immune response and reducing autoimmune responses (Fig. 3).

### The PD-1/PD-L1 pathway inhibits signal transduction in functional T cells

The mechanism by which PD-L1 inhibits the activation of T cell is the focus of investigation [47]. The activation of T cells requires signals from the T cell receptors (TCRs) and costimulatory receptors. After the initial activation of T cells, the complex formed from PD-L1 and PD-1 binding further inhibits signal transduction pathways in T cells, such as TCR signalling [41, 48], CD28 costimulatory signalling [39, 49], inducible costimulatory (ICOS) signalling [50], reducing the proliferation of self-reactive T cells.

The communication between TCR and its downstream molecules is required for T cell–APC interactions and the subsequent activation, proliferation, and differentiation of T cells. PD-1–PD-L1 binding was previously reported to attenuate interleukin 2 (IL-2) production and maintain peripheral tolerance by interfering with TCR-mediated stop signals [51]. After the activation of T cells, Src homology 2 domain-containing tyrosine phosphatase 2 (SHP2) becomes transiently recruited and associated with PD-1. This leads to the dephosphorylation of proximal TCR signalling molecules such as zeta chain of T cell receptor-associated protein kinase 70 (ZAP70), and the decreased phosphorylation of TCR downstream signalling molecules, such as the guanine nucleotide exchange factor Vav1, phospholipase C gamma 1 (PLCγ1), and protein kinase C theta (PKC theta), which are required for T-cell-mediated IL-2 production [48]. In addition, the modulation of T-cell function by PD-1–PD-L1 binding involves the inhibition of the phosphoinositide-3-kinase (PI3K) /AKT pathway and the Ras/mitogen-activated protein kinase (MEK)/extracellular signal-regulated kinase (ERK) pathway [41, 52], both of which are also involved in glucose metabolism. The inhibitory effect of PD-L1/PD-1 signalling helps regulate glycolysis and oxidative phosphorylation by reducing extracellular acidification and oxygen consumption rates, thereby affecting the metabolic reprogramming of activated primary T cells [53].

In an earlier study of the involvement of PD-L1 in the negative effect of lymphocytic PD-1, it was suggested that besides the inhibition of the TCR signalling pathway, the PD-L1/PD-1 signalling pathway could inhibit at least suboptimal levels of CD28-mediated co-stimulation [39]. In a recent study, researchers found dephosphorylated co-receptor CD28 in response to the activated PD-1/ PD-L1 pathway. Furthermore, they also suggested that CD28, not the TCR, was the most sensitive PD-1 target. It is well known that at high concentrations of PD-1, the TCR signalling components like ZAP70 will become dephosphorylated. However, through direct quantitative comparisons the researchers found that the dephosphorylation of TCR was always weaker than that of CD28. Collectively, these findings indicate that the main mechanism of PD-1-inhibits function of T cells is the inactivation of CD28 signalling [49]. Another study found that although the co-receptor CD28 could overcome the inactivation mediated by PD-1 by increasing IL-2 production, the binding of PD-1/PD-L1 also inhibits IL-2 production following co-stimulation. Obviously, after long-term activation, the dominant pathway remains the inhibitory pathway modulated by PD-1/PD-L1 [54].

The co-stimulatory pathway mediated by ICOS signalling is also sensitive to PD-1. Co-stimulation through ICOS could enhance T cell proliferation; however, this effect may be negligible in the presence of PD-1. The proliferative response induced by PD-1/ICOS ligand (ICOS-L) activation is analogous to that induced by anti-CD3 alone [50].

### The PD-1/PD-L1 pathway helps to sustain regulatory T cell function

Both the PD-1/PD-L1 pathway and Tregs are significant mediators of peripheral tolerance. Contrary to the inhibitory effects of PD-L1 on T cells, it has been demonstrated that PD-L1 can control the development of Tregs and sustain their function. In a study investigating the role of PD-L1 on the conversion of naïve CD4 T cells into induced regulatory T (iTreg) cells, researchers found that PD-L1 can induce iTreg cells in vitro, reduce iTreg cell conversion, and even enhance Foxp3 expression, maintaining the immunosuppressive effect of iTreg cells. As a transcription factor that is important in the regulatory activity of natural Treg (nTreg) and iTreg cells, the maintenance of Foxp3 expression may explain the functional role of PD-L1 in promoting the development of Tregs. Meanwhile, the levels of phosphorylation of Akt, mammalian target of rapamycin (mTOR), and ribosomal protein S6 were significantly decreased as PD-L1 levels increased, demonstrating that PD-L1 may also regulate the differentiation of Tregs by antagonising the Akt–mTOR pathway [45]. In addition, the PD-1-PD-L1 binding can promote the Treg-mediated inhibition of matrix metalloproteinase-9 (MMP-9) derived from neutrophils [55, 56]. Collectively, these findings suggest that PD-L1 can also exert inhibitory effects by promoting Tregs.

### The PD-1/PD-L1 pathway mediates the dysfunction of NK cells

Equally importantly, PD-1^+^ NK cells have been found in many tumour environments, such as ovarian carcinoma and head and neck cancer [46, 57]. After binding to PD-1, PD-L1 expressed in cancer cells reduce the NK cells response to tumours. NK cells induce tumour immunosurveillance by releasing chemokines and cytokines; the PD-1–PD-L1 axis precisely checks this process [58]. Interestingly, it has been found that PD-1^+^ NK cells induced PD-L1 expression on cancer cells by releasing IFN-γ, and the upregulated PD-1–PD-L1 axis suppressed the NK cell function. The PD-1–PD-L1 axis-mediated NK cell dysfunction largely depended on the induction of Tregs, which inhibit the process of T cells help NK cells by IL-2 [59, 60]. Many studies have shown that blocking PD-1 or PD-L1 can recover NK cells and enhanced their cytotoxicity against tumours with high PD-L1 expression [46, 59, 61].

Besides, the function of PD-L1^+^ NK cells not related to PD-1 have also been reported. Compared to PD-L1– NK cells, the cytokine production and cytotoxicity of PD-L1^+^ NK cells are significantly increased [62]. PD-L1 signalling can activates NK cells via the p38/NF-κB pathway, which further maintain NK cells cytotoxic and cytokine production.

Consequently, the combination of NK cells PD-1 and tumour cells PD-L1 inhibits the cytotoxic of NK cells against tumour, while cytotoxicity of PD-L1^+^ NK cells is significantly increased.

## The role of PD-L1 in different types of hypoxia-related diseases

### The potential role of PD-L1 in tumours

In tumour formation, the development of aberrant blood vessel structures generates a vascular system that often fails to meet the increasing demand for oxygen in rapidly enlarging tumours, resulting in the formation of a hypoxic environment in the tumour [63]. As an essential mediator expressed on hypoxic tumour cells, PD-L1 has an intriguing influence on tumour growth [64]. Previous studies have suggested that PD-L1 is constitutively expressed in various tumour cells, including those seen in haematological malignancies and metastatic melanomas. In the tumour microenvironment, the overexpression of PD-L1 is closely related to the increased expression of HIF-1α and the activation of NF-κB. Moreover, cytokines produced by activated immune cells such as TNF-α and IFN-γ can also upregulate the expression of PD-L1 [38, 41, 59]. The tumour microenvironment stimulates IFN-γ production by activated NK cells and further upregulates PD-L1 expression on the surface of tumour and NK cells [59, 62].Meanwhile, many exosomes are released from tumour cells, and stimulation with IFN-γ increases the amount of PD-L1 transported on their surface [43].

Overexpression of PD-L1 can facilitate tumour growth by inhibiting the function of CD8 T cells and promoting the function of Tregs. Firstly, the overexpression of PD-L1 can induce unresponsiveness or apoptosis of PD-1^+^ T cells by interfering with TCR signalling pathways. Meanwhile, the inhibition of TCR signalling also suppresses the production of TNF-α, IL-2, and IFN-γ. A few pharmacological agents have been confirmed to enhance T cell activity by inhibiting the expression of PD-L1 in cancer cells [65]. On the other hand, blocking PD-L1 directly limits the immunosuppressive capacity of Treg cells [66], indicating that the PD-L1/PD-1 pathway regulates the function of Tregs and participates in tumour-related immunosuppression. Furthermore, it has also been found that intrinsic PD-L1 signalling in tumours regulates cellular proliferation and autophagy in ovarian cancer and melanoma, and the attenuation of PD-L1 enhances autophagy and weakens the ability of autophagy inhibitors in NOD scid gamma (NSG) mice to restrict proliferation in vitro and in vivo [67]. This indicates an alternative role of anti-PD-L1 treatment in tumour immunotherapy.

Clinically, anti-PD-L1 antibodies have been highly efficacious in the treatment of certain tumours, such as metastatic melanomas, ovarian cancers, oesophageal squamous cell carcinomas, and haematopoietic malignancies [68–72]. Monoclonal antibodies (mAbs) have been used to reduce the immunosuppression of T cells, to increase the effector-to-suppressor cell ratio, and to support the maintenance of an anti-tumour microenvironment by preventing PD-L1 from associating with PD-1 [73, 74]. Moreover, PD-L1 expression can be considered as a biomarker of poor prognosis in cancer patients, and some studies have shown a role of PD-L1 in the induction of anti-tumour immune responses, contributing to better prognosis after surgery [75–77].

### The potential role of PD-L1 in stroke

Previously, we mentioned that the progressive deterioration following a stroke is closely related to neuroinflammation, which is caused by activated microglia and infiltrating T cells in the CNS. In chronic inflammation of the CNS, the PD-L1 expressed on microglia binds to PD-1 on the CD8^+^ T cells that persist in brain and negatively regulates the activation of T cells [78]. Similarly, after stroke, the overexpressed PD-L1 in microglia can restrict the severity of pathophysiological changes in the CNS and reduce acute ischaemic brain injury by reducing infiltrating T cells and inhibiting inflammatory cytokine production. We mentioned earlier that cytokines released by T cells, such as TNF-α and IFN-γ, can increase the number of PD-L1, which can modulate the function of T cells and reduce the secretion of pro-inflammatory cytokines. This inhibitory pathway may reduce the release of neurotoxic factors mediated by stroke-related Toll-like receptor 2 (TLR2) and TLR4 in activated microglia [31]. PD-L1 also significantly attenuates neurological deficits and reduces the volume of intracerebral haemorrhage (ICH) in mice by reducing brain-infiltrating CD4^+^ T cells and the proportions of Th1 and Th17 cells, while simultaneously increasing the percentages of Th2 and Tregs [79]. In addition, PD-L1 plays an essential role in neuroprotection by mediating the inhibition of Tregs on neutrophil-derived MMP-9 and by ameliorating the damage of blood–brain barrier after cerebral ischaemia [55]. However, in an experimental model of stroke, Offner et al. suggested that as the proportion of circulating PD-L1- and PD-L2-expressing CD19^+^ B cells increased in the periphery and CNS, increased levels of PD-1 limited the infarct volume through inhibiting the function of T cells and microglia; the findings implicate PD-1 signalling as a key factor in limiting CNS inflammation in murine experimental stroke models [31]. Subsequently, the group found that PD-L1 homozygous knock out (PD-L1-/-) mice had reduced levels of infiltrating CD4^+^ T cells in the ischaemic hemispheres, with smaller infarct volumes compared to those of wild-type (WT) mice, suggesting a pathogenic rather than a regulatory role for PD-L1 [80]. They suggested that this result may have been related to the overexpression of PD-1 and combining with PD-L2 after blocking PD-L1. Whether PD-L1 plays a positive role in attenuating neuroinflammation after cerebral ischaemia remains unknown. In summary, the PD-1/PD-L1 immunoregulatory pathway may be a new potential target for protecting against CNS injury in stroke in the future.

### The potential role of PD-L1 in OSAHS

OSAHS is characterised by repetitive episodes of intermittent hypoxaemia (IH). Studies have shown that OSAHS is associated with a higher incidence of cancer and a greater severity of infections due to immune dysregulation. To explore the relationship between IH and immunosuppression, researchers found upregulated PD-L1 expression both in vivo and in vitro [32, 81]. In a research involving patients with melanoma, soluble PD-L1 (sPD-L1) levels were found to be higher in patients with severe OSAHS than in those with mild OSAHS or non-OSAHS patients [82], indicating that the sPD-L1 concentration might be related to the degree of oxygen deficiency. Similarly, in an in vivo experiment, mice in the IH group exhibited high levels of PD-L1 expression compared to those in the control group following the injection of lung cancer cells into the flank and subsequent exposure to IH or normoxia for 1 week [83]. Therefore, the potential role of PD-L1 in disease and the therapeutic prospects of targeting the pathway have generated a great deal of interest from researchers. Ultimately, they found that upregulation of PD-L1 in OSAHS, which may be caused by higher HIF-1α activation, inhibits T cell proliferation and activation, and impairs the cytotoxic activity of CD8^+^ T cells [32], increasing the incidence and aggressiveness of certain cancers. The value of administering PD-L1 inhibitors in the treatment of OSAHS remains to be explored.

### The potential role of PD-L1 in AKI

The kidney is highly sensitive to blood flow shortages and hypoxia as a result of its high metabolic activity and vascular anatomy. Both innate and adaptive immune mechanisms participate in the pathophysiology of kidney ischemia–reperfusion injury (IRI), which is the leading cause of AKI. Mechanistically, it is well known that all Tregs have protective effects on renal function in AKI models by inhibiting innate immunity and modulating injury after kidney IRI [84–86]. Moreover, blocking the PD-L1/PD-1 pathway prior to mild renal IRI reverses the protective ability of Tregs, significantly aggravating renal dysfunction and acute tubular necrosis after ischaemia [87, 88]. In other words, PD-L1 may play a protective role in acute renal injury by promoting the inhibitory effects mediated by Tregs. Whether PD-L1 is involved in other innate and adaptive immune processes in AKI, such as those related to T cell function, remains to be further studied.

## Conclusions

In this review, we provided evidence that immune system dysfunction is a major cause of hypoxia-induced multiple organ injury. Immune surveillance functions become impaired in suppressed immune cells, making it difficult for the body to mount a response against viral infection and the rapid invasion of tumours. Excessive inflammatory reactions, on the other hand, are associated with protracted disease progression or secondary damage to the surrounding tissue. To explore the mechanisms of hypoxia-mediated immune dysfunction, we found that both persistent and intermittent hypoxia can directly trigger the overexpression of PD-L1 in various cell types and that these effects are closely related to HIF-1α activity. HIF-1α can regulate the expression of PD-L1 by directly acting on HRE-4 in the proximal promoter region of PD-L1. Consequently, PD‐L1 expressed in peripheral tissues helps reduce autoimmune damage and maintain peripheral tolerance through inhibiting T cells proliferation and promoting the differentiation of Tregs. Moreover, PD-L1 plays differential roles in various hypoxic diseases, and these findings indicate that the study of PD-L1 may lead to the discovery of powerful tools for diagnosing and treating these diseases. Therefore, further exploration of the role of PD-L1 in the pathophysiology and treatment of hypoxia-induced multiple organ injury is warranted in the future.

## Data Availability

Not applicable.

## Abbreviations

PD-L1: Programmed death-ligand 1
AKI: Acute kidney injury
OSAHS: Obstructive sleep apnoea hypopnoea syndrome
PD-1: Programmed cell death protein 1
Tregs: Regulatory T cells
COVID-19: Coronavirus disease 2019
NK: Natural killer
Th: Helper T cells
HIF: Hypoxia-inducible factor
FOXP3: Factor forkhead box P3
RORγt: RAR-related orphan receptor gamma
HMGB1: High-mobility group box protein 1
TLR4: Toll-like receptor 4
CNS: Central nervous system
NF-κB: Nuclear factor-κB
HRE-4: Hypoxia response element 4
IFN-γ: Interferon-γ
TNF-α: Tumour necrosis factor-α
APCs: Antigen-presenting cells
IL-10: Interleukin 10
IL-4: Interleukin 4
LPS: Lipopolysaccharide
TEXs: Tumour-derived exosomes
MDSCs: Myeloid-derived suppressor cells
γδT: Gamma delta T
TCRs: T cell receptors
ICOS: Inducible costimulatory
IL-2: Interleukin 2
SHP2: Src homology 2 domain-containing tyrosine phosphatase 2
ZAP70: Zeta chain of T cell receptor-associated protein kinase 70
PLCγ1: Phospholipase C gamma 1
PKCtheta: Protein kinase C theta
PI3K/AKT: Phosphoinositide-3-kinase
MEK: Mitogen-activated protein kinase
ERK: Extracellular signal-regulated kinase
ICOS-L: ICOS ligand
iTreg: Induced regulatory T
nTreg: Natural Treg
mTOR: Mammalian target of rapamycin
MMP-9: Matrix metalloproteinase-9
NSG: NOD scid gamma
mAbs: Monoclonal antibodies
TLR2: Toll-like receptor 2
ICH: Intracerebral haemorrhage
PD-L1-/-: PD-L1 homozygous knock out
WT: Wild-type
IH: Intermittent hypoxaemia
sPD-L1: Soluble PD-L1
IRI: Ischemia–reperfusion injury
